# Correction: Syed, T., et al. Current Insights on Vegetative Insecticidal Proteins (Vip) as Next Generation Pest Killers. *Toxins* 2020, *12*, 522

**DOI:** 10.3390/toxins13030200

**Published:** 2021-03-11

**Authors:** Tahira Syed, Muhammad Askari, Zhigang Meng, Yanyan Li, Muhammad Ali Abid, Yunxiao Wei, Sandui Guo, Chengzhen Liang, Rui Zhang

**Affiliations:** Biotechnology Research Institute, Chinese Academy of Agricultural Sciences, Beijing 100081, China; syedtahira98@gmail.com (T.S.); 2017Y90100082@caas.cn (M.A.); mengzhigang@caas.cn (Z.M.); liyanyan01@caas.cn (Y.L.); abid@caas.cn (M.A.A.); weiyunxiao@caas.cn (Y.W.); guosandui@caas.cn (S.G.)

The authors wish to make the following corrections to their paper [1]:

In the abstract and introduction section, the words “gram negative” should be replaced with “Gram positive” in first sentence.

In Section 6, the first line of the second paragraph, the second word should be replaced with “laboratory evolved resistance”. In the third sentence of same paragraph, reference 72 in the original paper should be replaced with reference [2] in this correction. 

In the third paragraph of this section, sentence five should be replaced with “Instead, a low proteolytic activity was found in resistant insects”. The last reference in this paragraph (reference 78 in the original paper) should be replaced by reference [3] in this correction.

In the fourth paragraph of this section, the first line should be replaced with “no cross resistance to Vip3C has been observed in insects of different species, previously found to be resistant to Cry1A, Cry2Ab, Dipel (Mixture of Cry1 and Cry2)”. The third sentence of this paragraph should be replaced with “The biochemical basis of resistance could not be established by the down regulation of membrane bound alkaline phosphatase (mALP) isoform HvmALP1, observed in Vip3 resistant insects, and results does not support it to be the functional receptor of Vip3”.

The reference in the second paragraph of Section 9.1 and the seventh row of Table 2 (reference 98 in the original paper) should be replaced by reference [4] in this correction.

We apologize for any inconvenience caused to readers of *Toxins* by this change. The manuscript will be updated and the original will remain online on the article webpage. We have also rearranged all references and citations according to the correct order. We apologize for any inconvenience caused to our readers.
